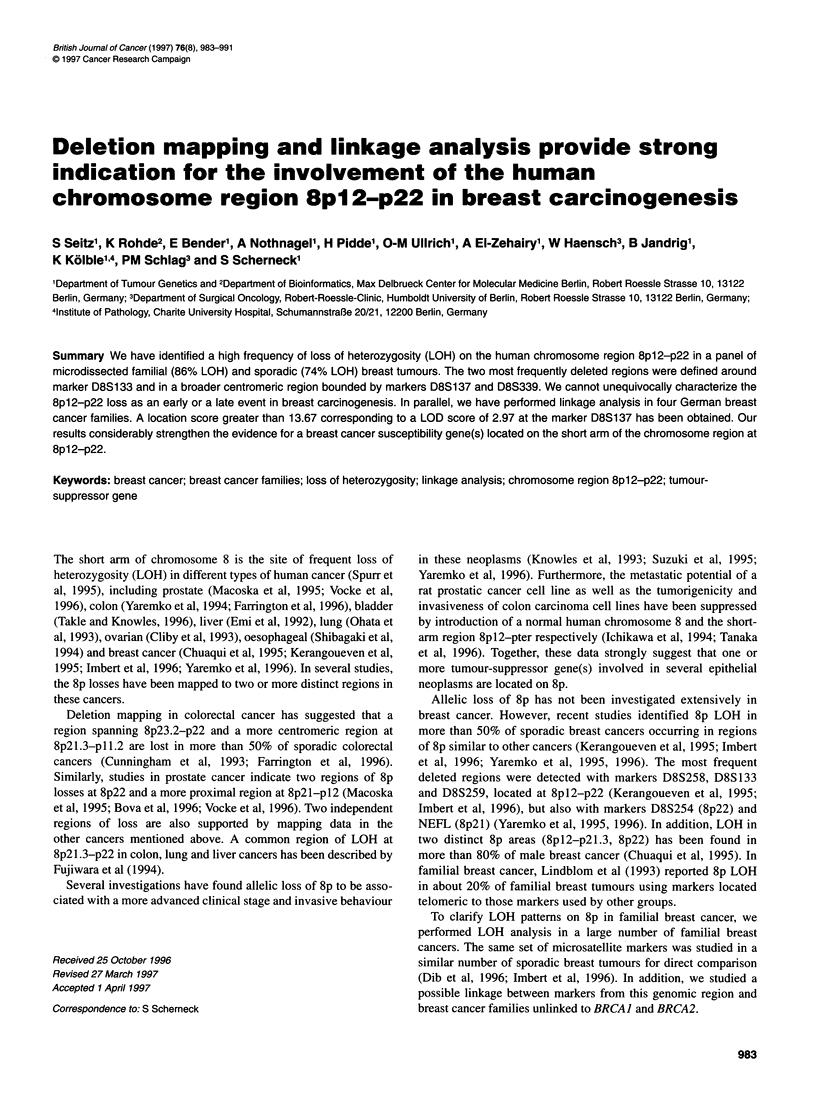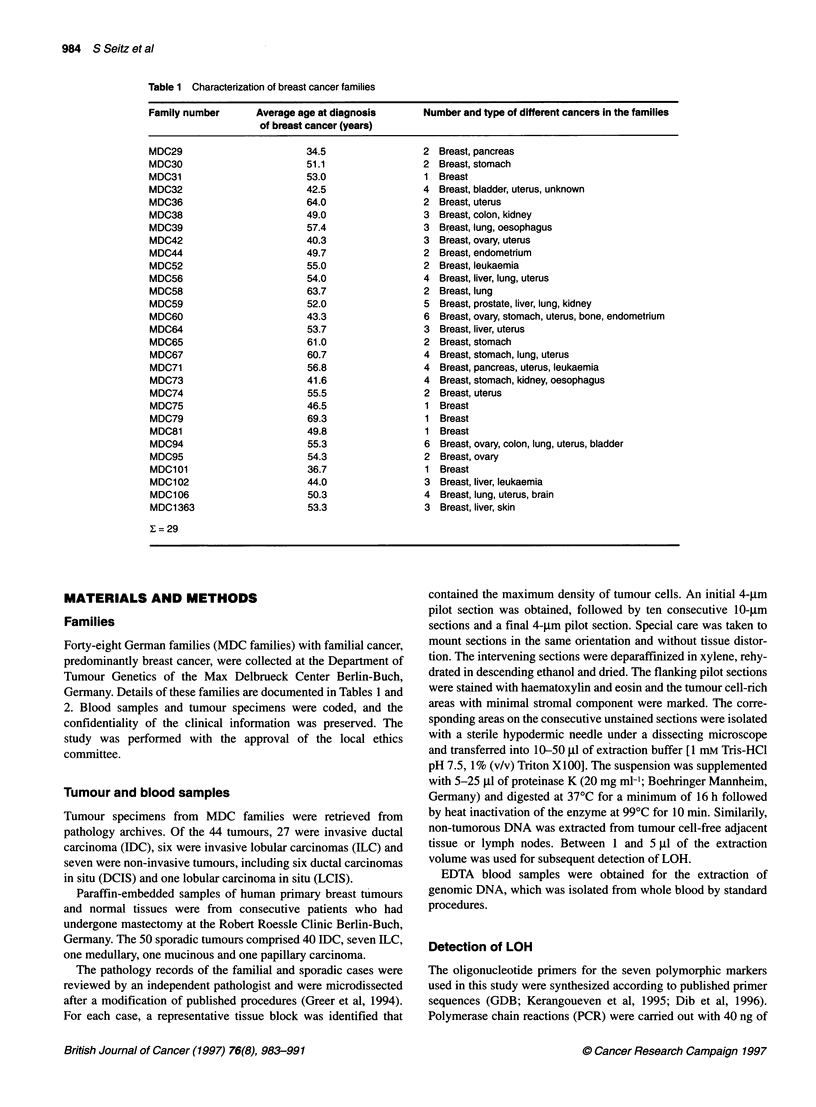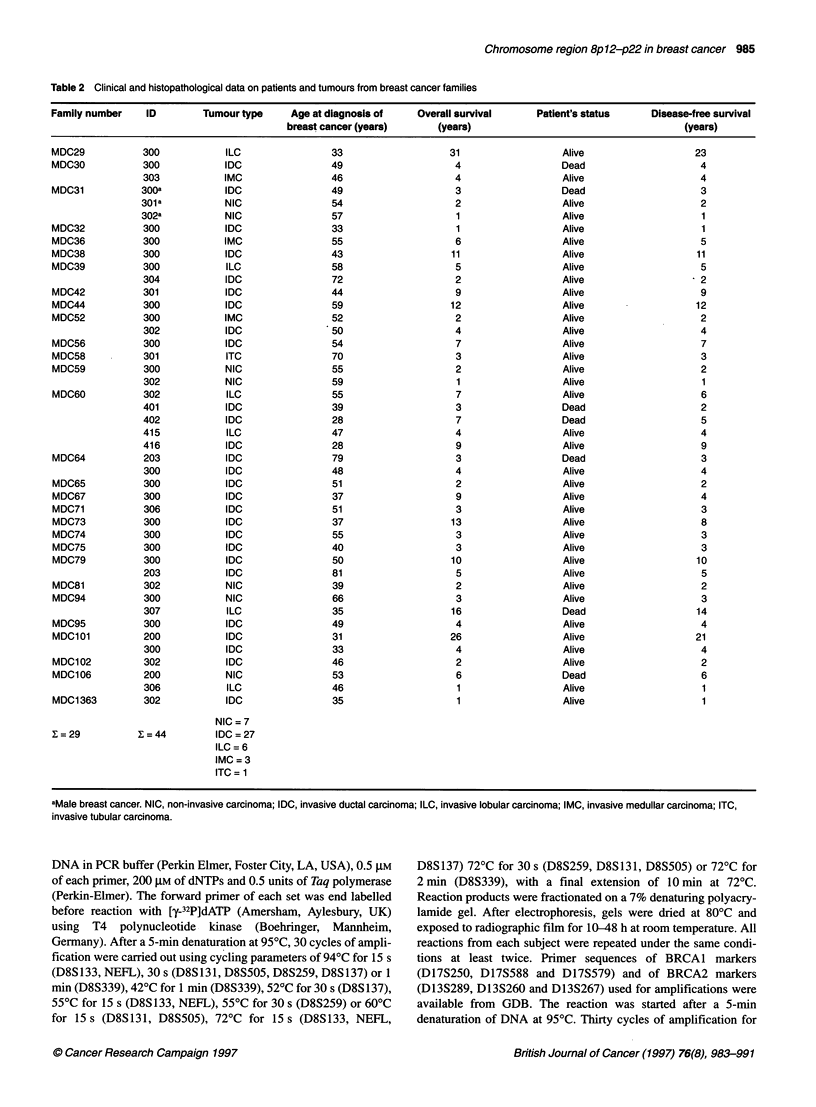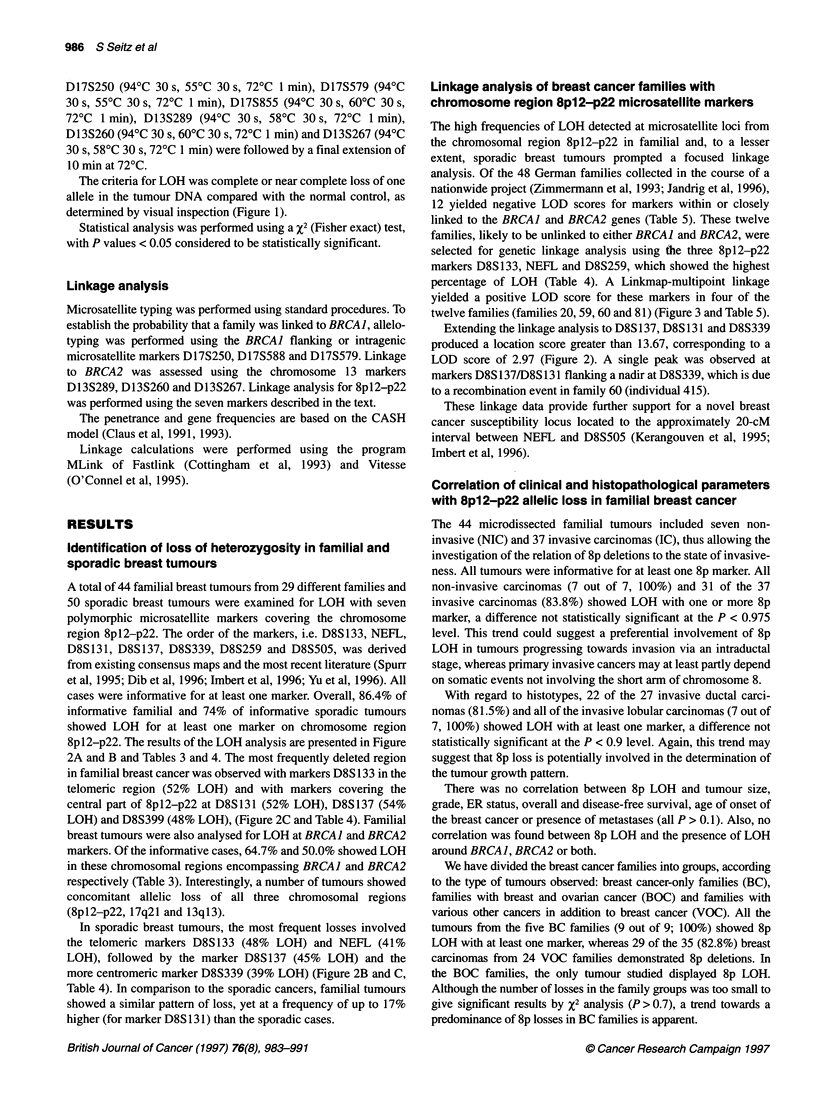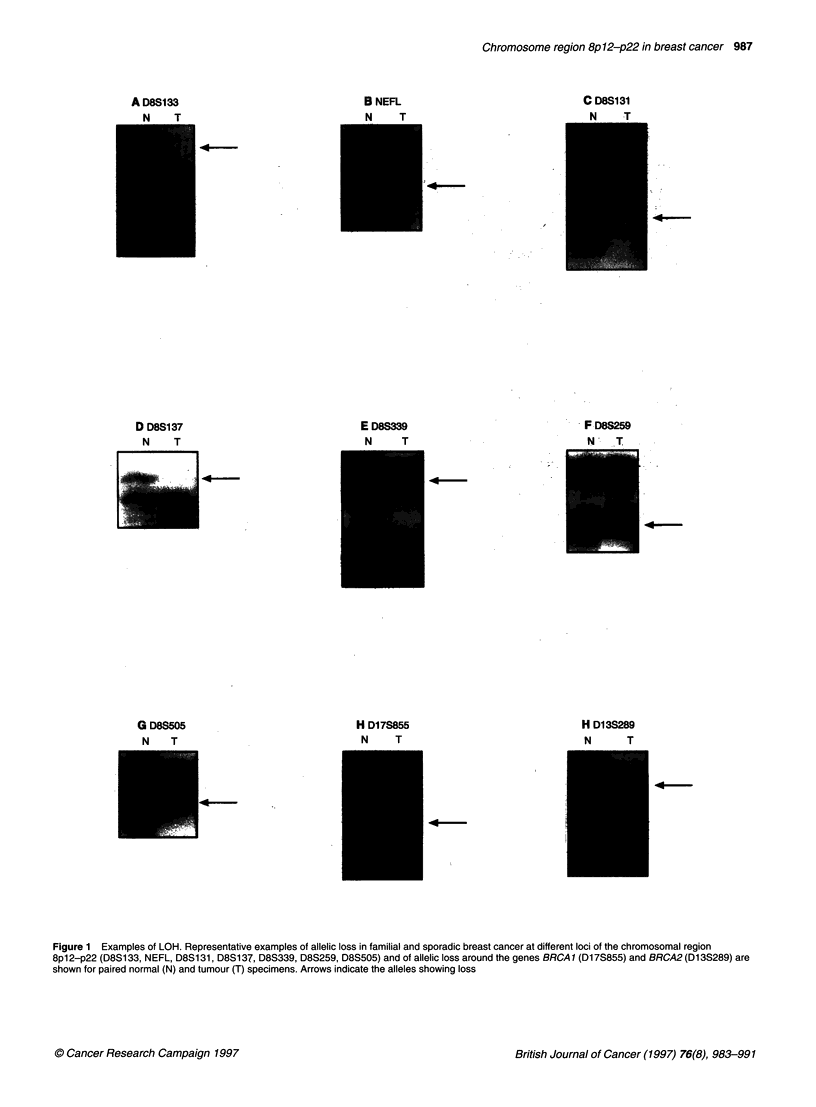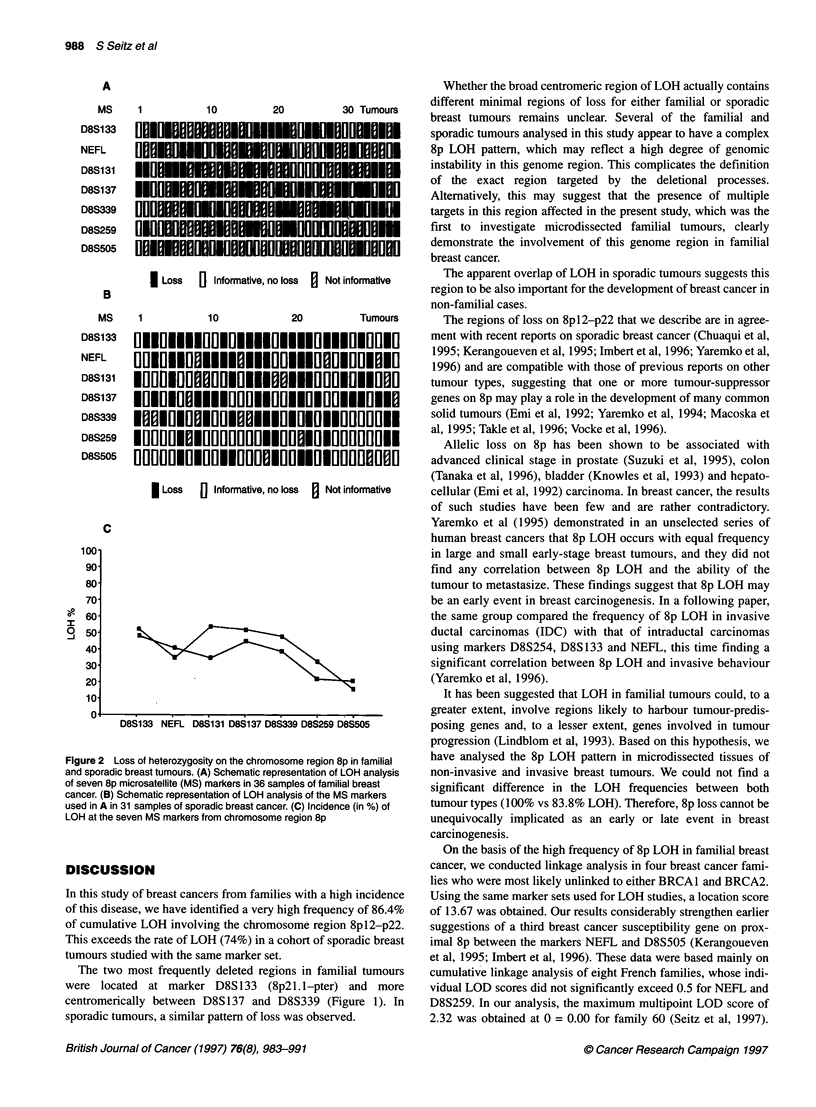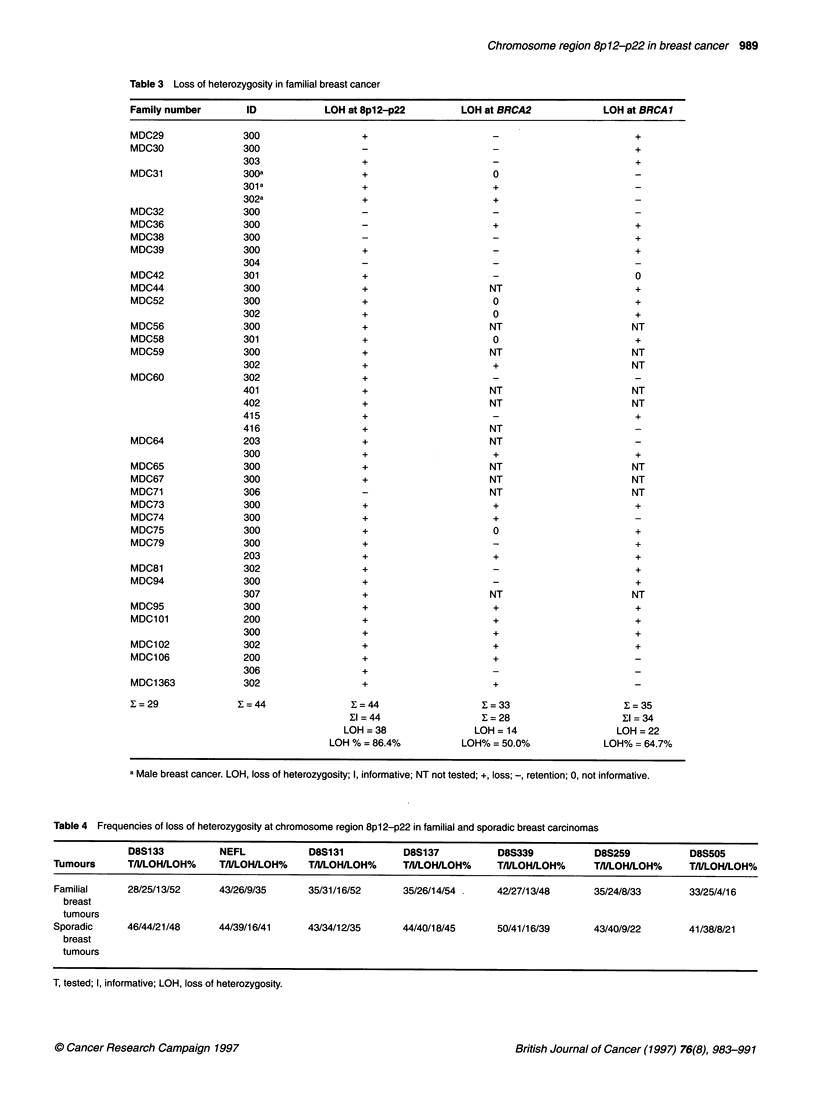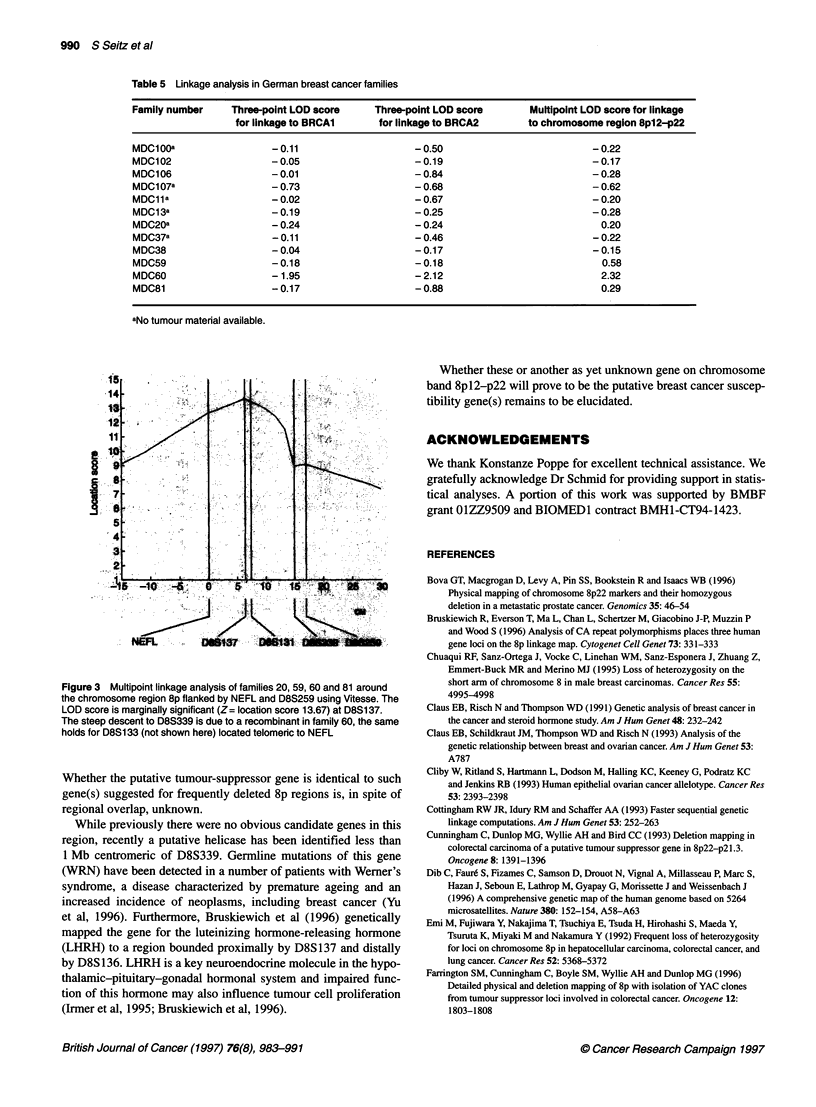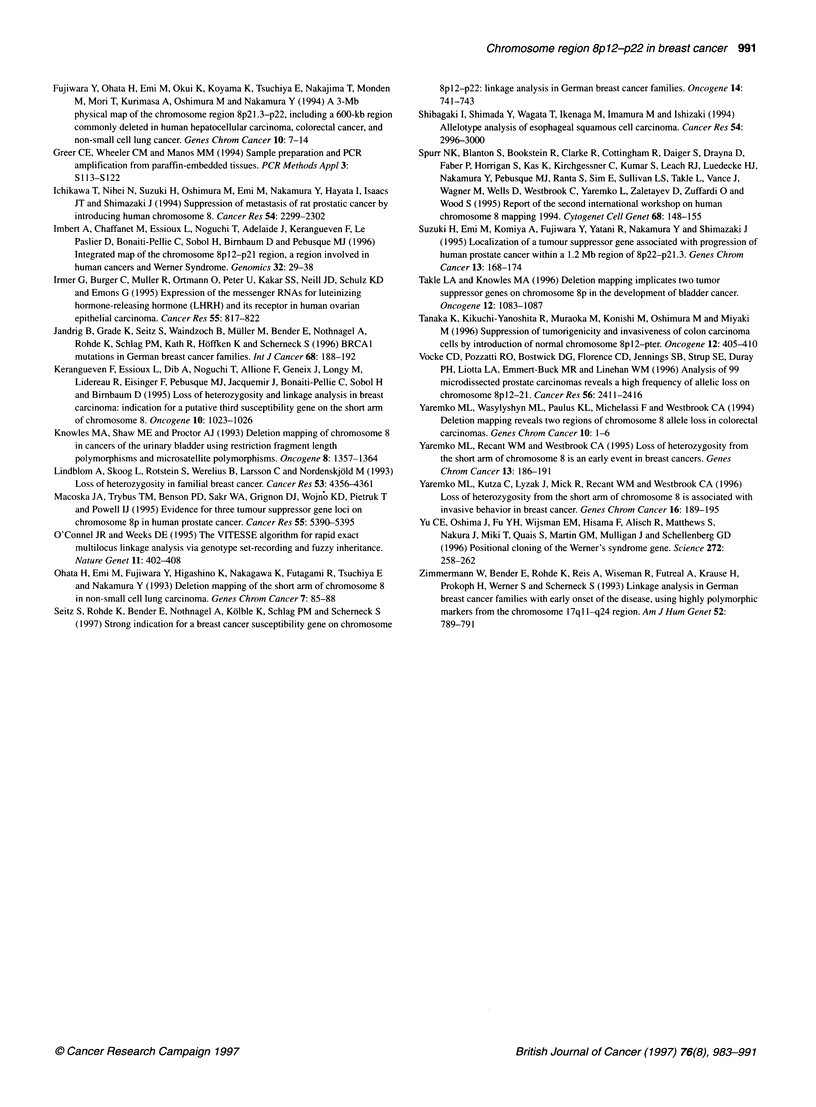# Deletion mapping and linkage analysis provide strong indication for the involvement of the human chromosome region 8p12-p22 in breast carcinogenesis.

**DOI:** 10.1038/bjc.1997.497

**Published:** 1997

**Authors:** S. Seitz, K. Rohde, E. Bender, A. Nothnagel, H. Pidde, O. M. Ullrich, A. El-Zehairy, W. Haensch, B. Jandrig, K. KÃ¶lble, P. M. Schlag, S. Scherneck

**Affiliations:** Department of Tumour Genetics, Max Delbrueck Center for Molecular Medicine Berlin, Germany.

## Abstract

**Images:**


British Journal of Cancer (1997) 76(8), 983–991
© 1997 Cancer Research Campaign

### Deletion mapping and linkage analysis provide strong indication for the involvement of the human chromosome region 8p12–p22 in breast carcinogenesis

**S Seitz[1], K Rohde[2], E Bender[1], A Nothnagel[1], H Pidde[1], O-M Ullrich[1], A El-Zehairy[1], W Haensch[3], B Jandrig[1], K Kölble[1,4], PM Schlag[3] and S Scherneck[1]**

[1]Department of Tumour Genetics and [2]Department of Bioinformatics, Max Delbrueck Center for Molecular Medicine Berlin, Robert Roessle Strasse 10, 13122 Berlin, Germany; [3]Department of Surgical Oncology, Robert-Roessle-Clinic, Humboldt University of Berlin, Robert Roessle Strasse 10, 13122 Berlin, Germany; [4]Institute of Pathology, Charite University Hospital, Schumannstraße 20/21, 12200 Berlin, Germany

**Summary** We have identified a high frequency of loss of heterozygosity (LOH) on the human chromosome region 8p12–p22 in a panel of microdissected familial (86% LOH) and sporadic (74% LOH) breast tumours. The two most frequently deleted regions were defined around marker D8S133 and in a broader centromeric region bounded by markers D8S137 and D8S339. We cannot unequivocally characterize the 8p12–p22 loss as an early or a late event in breast carcinogenesis. In parallel, we have performed linkage analysis in four German breast cancer families. A location score greater than 13.67 corresponding to a LOD score of 2.97 at the marker D8S137 has been obtained. Our results considerably strengthen the evidence for a breast cancer susceptibility gene(s) located on the short arm of the chromosome region at 8p12–p22.

**Keywords:** breast cancer; breast cancer families; loss of heterozygosity; linkage analysis; chromosome region 8p12–p22; tumour-suppressor gene

The short arm of chromosome 8 is the site of frequent loss of heterozygosity (LOH) in different types of human cancer (Spurr et al, 1995), including prostate (Macoska et al, 1995; Vocke et al, 1996), colon (Yaremko et al, 1994; Farrington et al, 1996), bladder (Takle and Knowles, 1996), liver (Emi et al, 1992), lung (Ohata et al, 1993), ovarian (Cliby et al, 1993), oesophageal (Shibagaki et al, 1994) and breast cancer (Chuaqui et al, 1995; Kerangoueven et al, 1995; Imbert et al, 1996; Yaremko et al, 1996). In several studies, the 8p losses have been mapped to two or more distinct regions in these cancers.

Deletion mapping in colorectal cancer has suggested that a region spanning 8p23.2–p22 and a more centromeric region at 8p21.3–p11.2 are lost in more than 50% of sporadic colorectal cancers (Cunningham et al, 1993; Farrington et al, 1996). Similarly, studies in prostate cancer indicate two regions of 8p losses at 8p22 and a more proximal region at 8p21–p12 (Macoska et al, 1995; Bova et al, 1996; Vocke et al, 1996). Two independent regions of loss are also supported by mapping data in the other cancers mentioned above. A common region of LOH at 8p21.3–p22 in colon, lung and liver cancers has been described by Fujiwara et al (1994).

Several investigations have found allelic loss of 8p to be associated with a more advanced clinical stage and invasive behaviour in these neoplasms (Knowles et al, 1993; Suzuki et al, 1995; Yaremko et al, 1996). Furthermore, the metastatic potential of a rat prostatic cancer cell line as well as the tumorigenicity and invasiveness of colon carcinoma cell lines have been suppressed by introduction of a normal human chromosome 8 and the short-arm region 8p12–pter respectively (Ichikawa et al, 1994; Tanaka et al, 1996). Together, these data strongly suggest that one or more tumour-suppressor gene(s) involved in several epithelial neoplasms are located on 8p.

Allelic loss of 8p has not been investigated extensively in breast cancer. However, recent studies identified 8p LOH in more than 50% of sporadic breast cancers occurring in regions of 8p similar to other cancers (Kerangoueven et al, 1995; Imbert et al, 1996; Yaremko et al, 1995, 1996). The most frequent deleted regions were detected with markers D8S258, D8S133 and D8S259, located at 8p12–p22 (Kerangoueven et al, 1995; Imbert et al, 1996), but also with markers D8S254 (8p22) and NEFL (8p21) (Yaremko et al, 1995, 1996). In addition, LOH in two distinct 8p areas (8p12–p21.3, 8p22) has been found in more than 80% of male breast cancer (Chuaqui et al, 1995). In familial breast cancer, Lindblom et al (1993) reported 8p LOH in about 20% of familial breast tumours using markers located telomeric to those markers used by other groups.

To clarify LOH patterns on 8p in familial breast cancer, we performed LOH analysis in a large number of familial breast cancers. The same set of microsatellite markers was studied in a similar number of sporadic breast tumours for direct comparison (Dib et al, 1996; Imbert et al, 1996). In addition, we studied a possible linkage between markers from this genomic region and breast cancer families unlinked to *BRCA1* and *BRCA2*.

*Received 25 October 1996*
*Revised 27 March 1997*
*Accepted 1 April 1997*

*Correspondence to:* S Scherneck

Table 1 Characterization of breast cancer families

| Family number | Average age at diagnosis of breast cancer (years) | Number and type of different cancers in the families |
|---|---|---|
| MDC29 | 34.5 | 2 Breast, pancreas |
| MDC30 | 51.1 | 2 Breast, stomach |
| MDC31 | 53.0 | 1 Breast |
| MDC32 | 42.5 | 4 Breast, bladder, uterus, unknown |
| MDC36 | 64.0 | 2 Breast, uterus |
| MDC38 | 49.0 | 3 Breast, colon, kidney |
| MDC39 | 57.4 | 3 Breast, lung, oesophagus |
| MDC42 | 40.3 | 3 Breast, ovary, uterus |
| MDC44 | 49.7 | 2 Breast, endometrium |
| MDC52 | 55.0 | 2 Breast, leukaemia |
| MDC56 | 54.0 | 4 Breast, liver, lung, uterus |
| MDC58 | 63.7 | 2 Breast, lung |
| MDC59 | 52.0 | 5 Breast, prostate, liver, lung, kidney |
| MDC60 | 43.3 | 6 Breast, ovary, stomach, uterus, bone, endometrium |
| MDC64 | 53.7 | 3 Breast, liver, uterus |
| MDC65 | 61.0 | 2 Breast, stomach |
| MDC67 | 60.7 | 4 Breast, stomach, lung, uterus |
| MDC71 | 56.8 | 4 Breast, pancreas, uterus, leukaemia |
| MDC73 | 41.6 | 4 Breast, stomach, kidney, oesophagus |
| MDC74 | 55.5 | 2 Breast, uterus |
| MDC75 | 46.5 | 1 Breast |
| MDC79 | 69.3 | 1 Breast |
| MDC81 | 49.8 | 1 Breast |
| MDC94 | 55.3 | 6 Breast, ovary, colon, lung, uterus, bladder |
| MDC95 | 54.3 | 2 Breast, ovary |
| MDC101 | 36.7 | 1 Breast |
| MDC102 | 44.0 | 3 Breast, liver, leukaemia |
| MDC106 | 50.3 | 4 Breast, lung, uterus, brain |
| MDC1363 | 53.3 | 3 Breast, liver, skin |
| Σ = 29 | | |

### MATERIALS AND METHODS

### Families

Forty-eight German families (MDC families) with familial cancer, predominantly breast cancer, were collected at the Department of Tumour Genetics of the Max Delbrueck Center Berlin-Buch, Germany. Details of these families are documented in Tables 1 and 2. Blood samples and tumour specimens were coded, and the confidentiality of the clinical information was preserved. The study was performed with the approval of the local ethics committee.

### Tumour and blood samples

Tumour specimens from MDC families were retrieved from pathology archives. Of the 44 tumours, 27 were invasive ductal carcinoma (IDC), six were invasive lobular carcinomas (ILC) and seven were non-invasive tumours, including six ductal carcinomas in situ (DCIS) and one lobular carcinoma in situ (LCIS).

Paraffin-embedded samples of human primary breast tumours and normal tissues were from consecutive patients who had undergone mastectomy at the Robert Roessle Clinic Berlin-Buch, Germany. The 50 sporadic tumours comprised 40 IDC, seven ILC, one medullary, one mucinous and one papillary carcinoma.

The pathology records of the familial and sporadic cases were reviewed by an independent pathologist and were microdissected after a modification of published procedures (Greer et al, 1994). For each case, a representative tissue block was identified that contained the maximum density of tumour cells. An initial 4-μm pilot section was obtained, followed by ten consecutive 10-μm sections and a final 4-μm pilot section. Special care was taken to mount sections in the same orientation and without tissue distortion. The intervening sections were deparaffinized in xylene, rehydrated in descending ethanol and dried. The flanking pilot sections were stained with haematoxylin and eosin and the tumour cell-rich areas with minimal stromal component were marked. The corresponding areas on the consecutive unstained sections were isolated with a sterile hypodermic needle under a dissecting microscope and transferred into 10–50 μl of extraction buffer [1 mM Tris-HCl pH 7.5, 1% (v/v) Triton X100]. The suspension was supplemented with 5–25 μl of proteinase K (20 mg $ml^{-1}$; Boehringer Mannheim, Germany) and digested at 37°C for a minimum of 16 h followed by heat inactivation of the enzyme at 99°C for 10 min. Similarily, non-tumorous DNA was extracted from tumour cell-free adjacent tissue or lymph nodes. Between 1 and 5 μl of the extraction volume was used for subsequent detection of LOH.

EDTA blood samples were obtained for the extraction of genomic DNA, which was isolated from whole blood by standard procedures.

### Detection of LOH

The oligonucleotide primers for the seven polymorphic markers used in this study were synthesized according to published primer sequences (GDB; Kerangoueven et al, 1995; Dib et al, 1996). Polymerase chain reactions (PCR) were carried out with 40 ng of

© Cancer Research Campaign 1997

**Table 2** Clinical and histopathological data on patients and tumours from breast cancer families

| Family number | ID | Tumour type | Age at diagnosis of breast cancer (years) | Overall survival (years) | Patient's status | Disease-free survival (years) |
|---|---|---|---|---|---|---|
| MDC29 | 300 | ILC | 33 | 31 | Alive | 23 |
| MDC30 | 300 | IDC | 49 | 4 | Dead | 4 |
| | 303 | IMC | 46 | 4 | Alive | 4 |
| MDC31 | 300[a] | IDC | 49 | 3 | Dead | 3 |
| | 301[a] | NIC | 54 | 2 | Alive | 2 |
| | 302[a] | NIC | 57 | 1 | Alive | 1 |
| MDC32 | 300 | IDC | 33 | 1 | Alive | 1 |
| MDC36 | 300 | IMC | 55 | 6 | Alive | 5 |
| MDC38 | 300 | IDC | 43 | 11 | Alive | 11 |
| MDC39 | 300 | ILC | 58 | 5 | Alive | 5 |
| | 304 | IDC | 72 | 2 | Alive | 2 |
| MDC42 | 301 | IDC | 44 | 9 | Alive | 9 |
| MDC44 | 300 | IDC | 59 | 12 | Alive | 12 |
| MDC52 | 300 | IMC | 52 | 2 | Alive | 2 |
| | 302 | IDC | 50 | 4 | Alive | 4 |
| MDC56 | 300 | IDC | 54 | 7 | Alive | 7 |
| MDC58 | 301 | ITC | 70 | 3 | Alive | 3 |
| MDC59 | 300 | NIC | 55 | 2 | Alive | 2 |
| | 302 | NIC | 59 | 1 | Alive | 1 |
| MDC60 | 302 | ILC | 55 | 7 | Alive | 6 |
| | 401 | IDC | 39 | 3 | Dead | 2 |
| | 402 | IDC | 28 | 7 | Dead | 5 |
| | 415 | ILC | 47 | 4 | Alive | 4 |
| | 416 | IDC | 28 | 9 | Alive | 9 |
| MDC64 | 203 | IDC | 79 | 3 | Dead | 3 |
| | 300 | IDC | 48 | 4 | Alive | 4 |
| MDC65 | 300 | IDC | 51 | 2 | Alive | 2 |
| MDC67 | 300 | IDC | 37 | 9 | Alive | 4 |
| MDC71 | 306 | IDC | 51 | 3 | Alive | 3 |
| MDC73 | 300 | IDC | 37 | 13 | Alive | 8 |
| MDC74 | 300 | IDC | 55 | 3 | Alive | 3 |
| MDC75 | 300 | IDC | 40 | 3 | Alive | 3 |
| MDC79 | 300 | IDC | 50 | 10 | Alive | 10 |
| | 203 | IDC | 81 | 5 | Alive | 5 |
| MDC81 | 302 | NIC | 39 | 2 | Alive | 2 |
| MDC94 | 300 | NIC | 66 | 3 | Alive | 3 |
| | 307 | ILC | 35 | 16 | Dead | 14 |
| MDC95 | 300 | IDC | 49 | 4 | Alive | 4 |
| MDC101 | 200 | IDC | 31 | 26 | Alive | 21 |
| | 300 | IDC | 33 | 4 | Alive | 4 |
| MDC102 | 302 | IDC | 46 | 2 | Alive | 2 |
| MDC106 | 200 | NIC | 53 | 6 | Dead | 6 |
| | 306 | ILC | 46 | 1 | Alive | 1 |
| MDC1363 | 302 | IDC | 35 | 1 | Alive | 1 |
| Σ = 29 | Σ = 44 | NIC = 7<br>IDC = 27<br>ILC = 6<br>IMC = 3<br>ITC = 1 | | | | |

[a]Male breast cancer. NIC, non-invasive carcinoma; IDC, invasive ductal carcinoma; ILC, invasive lobular carcinoma; IMC, invasive medullar carcinoma; ITC, invasive tubular carcinoma.

DNA in PCR buffer (Perkin Elmer, Foster City, LA, USA), 0.5 μM of each primer, 200 μM of dNTPs and 0.5 units of *Taq* polymerase (Perkin-Elmer). The forward primer of each set was end labelled before reaction with [γ-$^{32}$P]dATP (Amersham, Aylesbury, UK) using T4 polynucleotide kinase (Boehringer, Mannheim, Germany). After a 5-min denaturation at 95°C, 30 cycles of amplification were carried out using cycling parameters of 94°C for 15 s (D8S133, NEFL), 30 s (D8S131, D8S505, D8S259, D8S137) or 1 min (D8S339), 42°C for 1 min (D8S339), 52°C for 30 s (D8S137), 55°C for 15 s (D8S133, NEFL), 55°C for 30 s (D8S259) or 60°C for 15 s (D8S131, D8S505), 72°C for 15 s (D8S133, NEFL, D8S137) 72°C for 30 s (D8S259, D8S131, D8S505) or 72°C for 2 min (D8S339), with a final extension of 10 min at 72°C. Reaction products were fractionated on a 7% denaturing polyacrylamide gel. After electrophoresis, gels were dried at 80°C and exposed to radiographic film for 10–48 h at room temperature. All reactions from each subject were repeated under the same conditions at least twice. Primer sequences of BRCA1 markers (D17S250, D17S588 and D17S579) and of BRCA2 markers (D13S289, D13S260 and D13S267) used for amplifications were available from GDB. The reaction was started after a 5-min denaturation of DNA at 95°C. Thirty cycles of amplification for

D17S250 (94°C 30 s, 55°C 30 s, 72°C 1 min), D17S579 (94°C 30 s, 55°C 30 s, 72°C 1 min), D17S855 (94°C 30 s, 60°C 30 s, 72°C 1 min), D13S289 (94°C 30 s, 58°C 30 s, 72°C 1 min), D13S260 (94°C 30 s, 60°C 30 s, 72°C 1 min) and D13S267 (94°C 30 s, 58°C 30 s, 72°C 1 min) were followed by a final extension of 10 min at 72°C.

The criteria for LOH was complete or near complete loss of one allele in the tumour DNA compared with the normal control, as determined by visual inspection (Figure 1).

Statistical analysis was performed using a $\chi^2$ (Fisher exact) test, with $P$ values < 0.05 considered to be statistically significant.

### Linkage analysis

Microsatellite typing was performed using standard procedures. To establish the probability that a family was linked to *BRCA1*, allelotyping was performed using the *BRCA1* flanking or intragenic microsatellite markers D17S250, D17S588 and D17S579. Linkage to *BRCA2* was assessed using the chromosome 13 markers D13S289, D13S260 and D13S267. Linkage analysis for 8p12–p22 was performed using the seven markers described in the text.

The penetrance and gene frequencies are based on the CASH model (Claus et al, 1991, 1993).

Linkage calculations were performed using the program MLink of Fastlink (Cottingham et al, 1993) and Vitesse (O'Connel et al, 1995).

### RESULTS

### Identification of loss of heterozygosity in familial and sporadic breast tumours

A total of 44 familial breast tumours from 29 different families and 50 sporadic breast tumours were examined for LOH with seven polymorphic microsatellite markers covering the chromosome region 8p12–p22. The order of the markers, i.e. D8S133, NEFL, D8S131, D8S137, D8S339, D8S259 and D8S505, was derived from existing consensus maps and the most recent literature (Spurr et al, 1995; Dib et al, 1996; Imbert et al, 1996; Yu et al, 1996). All cases were informative for at least one marker. Overall, 86.4% of informative familial and 74% of informative sporadic tumours showed LOH for at least one marker on chromosome region 8p12–p22. The results of the LOH analysis are presented in Figure 2A and B and Tables 3 and 4. The most frequently deleted region in familial breast cancer was observed with markers D8S133 in the telomeric region (52% LOH) and with markers covering the central part of 8p12–p22 at D8S131 (52% LOH), D8S137 (54% LOH) and D8S399 (48% LOH), (Figure 2C and Table 4). Familial breast tumours were also analysed for LOH at *BRCA1* and *BRCA2* markers. Of the informative cases, 64.7% and 50.0% showed LOH in these chromosomal regions encompassing *BRCA1* and *BRCA2* respectively (Table 3). Interestingly, a number of tumours showed concomitant allelic loss of all three chromosomal regions (8p12–p22, 17q21 and 13q13).

In sporadic breast tumours, the most frequent losses involved the telomeric markers D8S133 (48% LOH) and NEFL (41% LOH), followed by the marker D8S137 (45% LOH) and the more centromeric marker D8S339 (39% LOH) (Figure 2B and C, Table 4). In comparison to the sporadic cancers, familial tumours showed a similar pattern of loss, yet at a frequency of up to 17% higher (for marker D8S131) than the sporadic cases.

### Linkage analysis of breast cancer families with chromosome region 8p12–p22 microsatellite markers

The high frequencies of LOH detected at microsatellite loci from the chromosomal region 8p12–p22 in familial and, to a lesser extent, sporadic breast tumours prompted a focused linkage analysis. Of the 48 German families collected in the course of a nationwide project (Zimmermann et al, 1993; Jandrig et al, 1996), 12 yielded negative LOD scores for markers within or closely linked to the *BRCA1* and *BRCA2* genes (Table 5). These twelve families, likely to be unlinked to either *BRCA1* and *BRCA2*, were selected for genetic linkage analysis using the three 8p12–p22 markers D8S133, NEFL and D8S259, which showed the highest percentage of LOH (Table 4). A Linkmap-multipoint linkage yielded a positive LOD score for these markers in four of the twelve families (families 20, 59, 60 and 81) (Figure 3 and Table 5).

Extending the linkage analysis to D8S137, D8S131 and D8S339 produced a location score greater than 13.67, corresponding to a LOD score of 2.97 (Figure 2). A single peak was observed at markers D8S137/D8S131 flanking a nadir at D8S339, which is due to a recombination event in family 60 (individual 415).

These linkage data provide further support for a novel breast cancer susceptibility locus located to the approximately 20-cM interval between NEFL and D8S505 (Kerangouven et al, 1995; Imbert et al, 1996).

### Correlation of clinical and histopathological parameters with 8p12–p22 allelic loss in familial breast cancer

The 44 microdissected familial tumours included seven non-invasive (NIC) and 37 invasive carcinomas (IC), thus allowing the investigation of the relation of 8p deletions to the state of invasiveness. All tumours were informative for at least one 8p marker. All non-invasive carcinomas (7 out of 7, 100%) and 31 of the 37 invasive carcinomas (83.8%) showed LOH with one or more 8p marker, a difference not statistically significant at the $P < 0.975$ level. This trend could suggest a preferential involvement of 8p LOH in tumours progressing towards invasion via an intraductal stage, whereas primary invasive cancers may at least partly depend on somatic events not involving the short arm of chromosome 8.

With regard to histotypes, 22 of the 27 invasive ductal carcinomas (81.5%) and all of the invasive lobular carcinomas (7 out of 7, 100%) showed LOH with at least one marker, a difference not statistically significant at the $P < 0.9$ level. Again, this trend may suggest that 8p loss is potentially involved in the determination of the tumour growth pattern.

There was no correlation between 8p LOH and tumour size, grade, ER status, overall and disease-free survival, age of onset of the breast cancer or presence of metastases (all $P > 0.1$). Also, no correlation was found between 8p LOH and the presence of LOH around *BRCA1*, *BRCA2* or both.

We have divided the breast cancer families into groups, according to the type of tumours observed: breast cancer-only families (BC), families with breast and ovarian cancer (BOC) and families with various other cancers in addition to breast cancer (VOC). All the tumours from the five BC families (9 out of 9; 100%) showed 8p LOH with at least one marker, whereas 29 of the 35 (82.8%) breast carcinomas from 24 VOC families demonstrated 8p deletions. In the BOC families, the only tumour studied displayed 8p LOH. Although the number of losses in the family groups was too small to give significant results by $\chi^2$ analysis ($P > 0.7$), a trend towards a predominance of 8p losses in BC families is apparent.

© Cancer Research Campaign 1997

**Figure 1** Examples of LOH. Representative examples of allelic loss in familial and sporadic breast cancer at different loci of the chromosomal region 8p12–p22 (D8S133, NEFL, D8S131, D8S137, D8S339, D8S259, D8S505) and of allelic loss around the genes *BRCA1* (D17S855) and *BRCA2* (D13S289) are shown for paired normal (N) and tumour (T) specimens. Arrows indicate the alleles showing loss

**Figure 2** Loss of heterozygosity on the chromosome region 8p in familial and sporadic breast tumours. (**A**) Schematic representation of LOH analysis of seven 8p microsatellite (MS) markers in 36 samples of familial breast cancer. (**B**) Schematic representation of LOH analysis of the MS markers used in **A** in 31 samples of sporadic breast cancer. (**C**) Incidence (in %) of LOH at the seven MS markers from chromosome region 8p

### DISCUSSION

In this study of breast cancers from families with a high incidence of this disease, we have identified a very high frequency of 86.4% of cumulative LOH involving the chromosome region 8p12–p22. This exceeds the rate of LOH (74%) in a cohort of sporadic breast tumours studied with the same marker set.

The two most frequently deleted regions in familial tumours were located at marker D8S133 (8p21.1–pter) and more centromerically between D8S137 and D8S339 (Figure 1). In sporadic tumours, a similar pattern of loss was observed.

Whether the broad centromeric region of LOH actually contains different minimal regions of loss for either familial or sporadic breast tumours remains unclear. Several of the familial and sporadic tumours analysed in this study appear to have a complex 8p LOH pattern, which may reflect a high degree of genomic instability in this genome region. This complicates the definition of the exact region targeted by the deletional processes. Alternatively, this may suggest that the presence of multiple targets in this region affected in the present study, which was the first to investigate microdissected familial tumours, clearly demonstrate the involvement of this genome region in familial breast cancer.

The apparent overlap of LOH in sporadic tumours suggests this region to be also important for the development of breast cancer in non-familial cases.

The regions of loss on 8p12–p22 that we describe are in agreement with recent reports on sporadic breast cancer (Chuaqui et al, 1995; Kerangoueven et al, 1995; Imbert et al, 1996; Yaremko et al, 1996) and are compatible with those of previous reports on other tumour types, suggesting that one or more tumour-suppressor genes on 8p may play a role in the development of many common solid tumours (Emi et al, 1992; Yaremko et al, 1994; Macoska et al, 1995; Takle et al, 1996; Vocke et al, 1996).

Allelic loss on 8p has been shown to be associated with advanced clinical stage in prostate (Suzuki et al, 1995), colon (Tanaka et al, 1996), bladder (Knowles et al, 1993) and hepatocellular (Emi et al, 1992) carcinoma. In breast cancer, the results of such studies have been few and are rather contradictory. Yaremko et al (1995) demonstrated in an unselected series of human breast cancers that 8p LOH occurs with equal frequency in large and small early-stage breast tumours, and they did not find any correlation between 8p LOH and the ability of the tumour to metastasize. These findings suggest that 8p LOH may be an early event in breast carcinogenesis. In a following paper, the same group compared the frequency of 8p LOH in invasive ductal carcinomas (IDC) with that of intraductal carcinomas using markers D8S254, D8S133 and NEFL, this time finding a significant correlation between 8p LOH and invasive behaviour (Yaremko et al, 1996).

It has been suggested that LOH in familial tumours could, to a greater extent, involve regions likely to harbour tumour-predisposing genes and, to a lesser extent, genes involved in tumour progression (Lindblom et al, 1993). Based on this hypothesis, we have analysed the 8p LOH pattern in microdissected tissues of non-invasive and invasive breast tumours. We could not find a significant difference in the LOH frequencies between both tumour types (100% vs 83.8% LOH). Therefore, 8p loss cannot be unequivocally implicated as an early or late event in breast carcinogenesis.

On the basis of the high frequency of 8p LOH in familial breast cancer, we conducted linkage analysis in four breast cancer families who were most likely unlinked to either BRCA1 and BRCA2. Using the same marker sets used for LOH studies, a location score of 13.67 was obtained. Our results considerably strengthen earlier suggestions of a third breast cancer susceptibility gene on proximal 8p between the markers NEFL and D8S505 (Kerangoueven et al, 1995; Imbert et al, 1996). These data were based mainly on cumulative linkage analysis of eight French families, whose individual LOD scores did not significantly exceed 0.5 for NEFL and D8S259. In our analysis, the maximum multipoint LOD score of 2.32 was obtained at $0 = 0.00$ for family 60 (Seitz et al, 1997).

© Cancer Research Campaign 1997

**Table 3** Loss of heterozygosity in familial breast cancer

| Family number | ID | LOH at 8p12–p22 | LOH at *BRCA2* | LOH at *BRCA1* |
|---|---|---|---|---|
| MDC29 | 300 | + | – | + |
| MDC30 | 300 | – | – | + |
| | 303 | + | – | + |
| MDC31 | 300[a] | + | 0 | – |
| | 301[a] | + | + | – |
| | 302[a] | + | + | – |
| MDC32 | 300 | – | – | – |
| MDC36 | 300 | – | + | + |
| MDC38 | 300 | – | – | + |
| MDC39 | 300 | + | – | + |
| | 304 | – | – | – |
| MDC42 | 301 | + | – | 0 |
| MDC44 | 300 | + | NT | + |
| MDC52 | 300 | + | 0 | + |
| | 302 | + | 0 | + |
| MDC56 | 300 | + | NT | NT |
| MDC58 | 301 | + | 0 | + |
| MDC59 | 300 | + | NT | NT |
| | 302 | + | + | NT |
| MDC60 | 302 | + | – | – |
| | 401 | + | NT | NT |
| | 402 | + | NT | NT |
| | 415 | + | – | + |
| | 416 | + | NT | – |
| MDC64 | 203 | + | NT | – |
| | 300 | + | + | + |
| MDC65 | 300 | + | NT | NT |
| MDC67 | 300 | + | NT | NT |
| MDC71 | 306 | – | NT | NT |
| MDC73 | 300 | + | + | + |
| MDC74 | 300 | + | + | – |
| MDC75 | 300 | + | 0 | + |
| MDC79 | 300 | + | – | + |
| | 203 | + | + | + |
| MDC81 | 302 | + | – | + |
| MDC94 | 300 | + | – | + |
| | 307 | + | NT | NT |
| MDC95 | 300 | + | + | + |
| MDC101 | 200 | + | + | + |
| | 300 | + | + | + |
| MDC102 | 302 | + | + | + |
| MDC106 | 200 | + | + | – |
| | 306 | + | – | – |
| MDC1363 | 302 | + | + | – |
| Σ = 29 | Σ = 44 | Σ = 44<br>ΣI = 44<br>LOH = 38<br>LOH % = 86.4% | Σ = 33<br>Σ = 28<br>LOH = 14<br>LOH% = 50.0% | Σ = 35<br>ΣI = 34<br>LOH = 22<br>LOH% = 64.7% |

[a] Male breast cancer. LOH, loss of heterozygosity; I, informative; NT not tested; +, loss; –, retention; 0, not informative.

**Table 4** Frequencies of loss of heterozygosity at chromosome region 8p12–p22 in familial and sporadic breast carcinomas

| Tumours | D8S133 T/I/LOH/LOH% | NEFL T/I/LOH/LOH% | D8S131 T/I/LOH/LOH% | D8S137 T/I/LOH/LOH% | D8S339 T/I/LOH/LOH% | D8S259 T/I/LOH/LOH% | D8S505 T/I/LOH/LOH% |
|---|---|---|---|---|---|---|---|
| Familial breast tumours | 28/25/13/52 | 43/26/9/35 | 35/31/16/52 | 35/26/14/54 | 42/27/13/48 | 35/24/8/33 | 33/25/4/16 |
| Sporadic breast tumours | 46/44/21/48 | 44/39/16/41 | 43/34/12/35 | 44/40/18/45 | 50/41/16/39 | 43/40/9/22 | 41/38/8/21 |

T, tested; I, informative; LOH, loss of heterozygosity.

**Table 5** Linkage analysis in German breast cancer families

| Family number | Three-point LOD score for linkage to BRCA1 | Three-point LOD score for linkage to BRCA2 | Multipoint LOD score for linkage to chromosome region 8p12–p22 |
|---|---|---|---|
| MDC100[a] | −0.11 | −0.50 | −0.22 |
| MDC102 | −0.05 | −0.19 | −0.17 |
| MDC106 | −0.01 | −0.84 | −0.28 |
| MDC107[a] | −0.73 | −0.68 | −0.62 |
| MDC11[a] | −0.02 | −0.67 | −0.20 |
| MDC13[a] | −0.19 | −0.25 | −0.28 |
| MDC20[a] | −0.24 | −0.24 | 0.20 |
| MDC37[a] | −0.11 | −0.46 | −0.22 |
| MDC38 | −0.04 | −0.17 | −0.15 |
| MDC59 | −0.18 | −0.18 | 0.58 |
| MDC60 | −1.95 | −2.12 | 2.32 |
| MDC81 | −0.17 | −0.88 | 0.29 |

[a]No tumour material available.

**Figure 3** Multipoint linkage analysis of families 20, 59, 60 and 81 around the chromosome region 8p flanked by NEFL and D8S259 using Vitesse. The LOD score is marginally significant ($Z$ = location score 13.67) at D8S137. The steep descent to D8S339 is due to a recombinant in family 60, the same holds for D8S133 (not shown here) located telomeric to NEFL

Whether the putative tumour-suppressor gene is identical to such gene(s) suggested for frequently deleted 8p regions is, in spite of regional overlap, unknown.

While previously there were no obvious candidate genes in this region, recently a putative helicase has been identified less than 1 Mb centromeric of D8S339. Germline mutations of this gene (WRN) have been detected in a number of patients with Werner's syndrome, a disease characterized by premature ageing and an increased incidence of neoplasms, including breast cancer (Yu et al, 1996). Furthermore, Bruskiewich et al (1996) genetically mapped the gene for the luteinizing hormone-releasing hormone (LHRH) to a region bounded proximally by D8S137 and distally by D8S136. LHRH is a key neuroendocrine molecule in the hypothalamic–pituitary–gonadal hormonal system and impaired function of this hormone may also influence tumour cell proliferation (Irmer et al, 1995; Bruskiewich et al, 1996).

Whether these or another as yet unknown gene on chromosome band 8p12–p22 will prove to be the putative breast cancer susceptibility gene(s) remains to be elucidated.

### ACKNOWLEDGEMENTS

We thank Konstanze Poppe for excellent technical assistance. We gratefully acknowledge Dr Schmid for providing support in statistical analyses. A portion of this work was supported by BMBF grant 01ZZ9509 and BIOMED1 contract BMH1-CT94-1423.

### REFERENCES

Bova GT, Macgrogan D, Levy A, Pin SS, Bookstein R and Isaacs WB (1996) Physical mapping of chromosome 8p22 markers and their homozygous deletion in a metastatic prostate cancer. *Genomics* **35**: 46–54

Bruskiewich R, Everson T, Ma L, Chan L, Schertzer M, Giacobino J-P, Muzzin P and Wood S (1996) Analysis of CA repeat polymorphisms places three human gene loci on the 8p linkage map. *Cytogenet Cell Genet* **73**: 331–333

Chuaqui RF, Sanz-Ortega J, Vocke C, Linehan WM, Sanz-Esponera J, Zhuang Z, Emmert-Buck MR and Merino MJ (1995) Loss of heterozygosity on the short arm of chromosome 8 in male breast carcinomas. *Cancer Res* **55**: 4995–4998

Claus EB, Risch N and Thompson WD (1991) Genetic analysis of breast cancer in the cancer and steroid hormone study. *Am J Hum Genet* **48**: 232–242

Claus EB, Schildkraut JM, Thompson WD and Risch N (1993) Analysis of the genetic relationship between breast and ovarian cancer. *Am J Hum Genet* **53**: A787

Cliby W, Ritland S, Hartmann L, Dodson M, Halling KC, Keeney G, Podratz KC and Jenkins RB (1993) Human epithelial ovarian cancer allelotype. *Cancer Res* **53**: 2393–2398

Cottingham RW JR, Idury RM and Schaffer AA (1993) Faster sequential genetic linkage computations. *Am J Hum Genet* **53**: 252–263

Cunningham C, Dunlop MG, Wyllie AH and Bird CC (1993) Deletion mapping in colorectal carcinoma of a putative tumour suppressor gene in 8p22–p21.3. *Oncogene* **8**: 1391–1396

Dib C, Fauré S, Fizames C, Samson D, Drouot N, Vignal A, Millasseau P, Marc S, Hazan J, Seboun E, Lathrop M, Gyapay G, Morissette J and Weissenbach J (1996) A comprehensive genetic map of the human genome based on 5264 microsatellites. *Nature* **380**: 152–154, A58–A63

Emi M, Fujiwara Y, Nakajima T, Tsuchiya E, Tsuda H, Hirohashi S, Maeda Y, Tsuruta K, Miyaki M and Nakamura Y (1992) Frequent loss of heterozygosity for loci on chromosome 8p in hepatocellular carcinoma, colorectal cancer, and lung cancer. *Cancer Res* **52**: 5368–5372

Farrington SM, Cunningham C, Boyle SM, Wyllie AH and Dunlop MG (1996) Detailed physical and deletion mapping of 8p with isolation of YAC clones from tumour suppressor loci involved in colorectal cancer. *Oncogene* **12**: 1803–1808

 © Cancer Research Campaign 1997

Fujiwara Y, Ohata H, Emi M, Okui K, Koyama K, Tsuchiya E, Nakajima T, Monden M, Mori T, Kurimasa A, Oshimura M and Nakamura Y (1994) A 3-Mb physical map of the chromosome region 8p21.3–p22, including a 600-kb region commonly deleted in human hepatocellular carcinoma, colorectal cancer, and non-small cell lung cancer. *Genes Chrom Cancer* **10**: 7–14

Greer CE, Wheeler CM and Manos MM (1994) Sample preparation and PCR amplification from paraffin-embedded tissues. *PCR Methods Appl* **3**: S113–S122

Ichikawa T, Nihei N, Suzuki H, Oshimura M, Emi M, Nakamura Y, Hayata I, Isaacs JT and Shimazaki J (1994) Suppression of metastasis of rat prostatic cancer by introducing human chromosome 8. *Cancer Res* **54**: 2299–2302

Imbert A, Chaffanet M, Essioux L, Noguchi T, Adelaide J, Kerangueven F, Le Paslier D, Bonaiti-Pellie C, Sobol H, Birnbaum D and Pebusque MJ (1996) Integrated map of the chromosome 8p12–p21 region, a region involved in human cancers and Werner Syndrome. *Genomics* **32**: 29–38

Irmer G, Burger C, Muller R, Ortmann O, Peter U, Kakar SS, Neill JD, Schulz KD and Emons G (1995) Expression of the messenger RNAs for luteinizing hormone-releasing hormone (LHRH) and its receptor in human ovarian epithelial carcinoma. *Cancer Res* **55**: 817–822

Jandrig B, Grade K, Seitz S, Waindzoch B, Müller M, Bender E, Nothnagel A, Rohde K, Schlag PM, Kath R, Höffken K and Scherneck S (1996) BRCA1 mutations in German breast cancer families. *Int J Cancer* **68**: 188–192

Kerangueven F, Essioux L, Dib A, Noguchi T, Allione F, Geneix J, Longy M, Lidereau R, Eisinger F, Pebusque MJ, Jacquemir J, Bonaiti-Pellie C, Sobol H and Birnbaum D (1995) Loss of heterozygosity and linkage analysis in breast carcinoma: indication for a putative third susceptibility gene on the short arm of chromosome 8. *Oncogene* **10**: 1023–1026

Knowles MA, Shaw ME and Proctor AJ (1993) Deletion mapping of chromosome 8 in cancers of the urinary bladder using restriction fragment length polymorphisms and microsatellite polymorphisms. *Oncogene* **8**: 1357–1364

Lindblom A, Skoog L, Rotstein S, Werelius B, Larsson C and Nordenskjöld M (1993) Loss of heterozygosity in familial breast cancer. *Cancer Res* **53**: 4356–4361

Macoska JA, Trybus TM, Benson PD, Sakr WA, Grignon DJ, Wojno KD, Pietruk T and Powell IJ (1995) Evidence for three tumour suppressor gene loci on chromosome 8p in human prostate cancer. *Cancer Res* **55**: 5390–5395

O'Connel JR and Weeks DE (1995) The VITESSE algorithm for rapid exact multilocus linkage analysis via genotype set-recording and fuzzy inheritance. *Nature Genet* **11**: 402–408

Ohata H, Emi M, Fujiwara Y, Higashino K, Nakagawa K, Futagami R, Tsuchiya E and Nakamura Y (1993) Deletion mapping of the short arm of chromosome 8 in non-small cell lung carcinoma. *Genes Chrom Cancer* **7**: 85–88

Seitz S, Rohde K, Bender E, Nothnagel A, Kölble K, Schlag PM and Scherneck S (1997) Strong indication for a breast cancer susceptibility gene on chromosome 8p12–p22: linkage analysis in German breast cancer families. *Oncogene* **14**: 741–743

Shibagaki I, Shimada Y, Wagata T, Ikenaga M, Imamura M and Ishizaki (1994) Allelotype analysis of esophageal squamous cell carcinoma. *Cancer Res* **54**: 2996–3000

Spurr NK, Blanton S, Bookstein R, Clarke R, Cottingham R, Daiger S, Drayna D, Faber P, Horrigan S, Kas K, Kirchgessner C, Kumar S, Leach RJ, Luedecke HJ, Nakamura Y, Pebusque MJ, Ranta S, Sim E, Sullivan LS, Takle L, Vance J, Wagner M, Wells D, Westbrook C, Yaremko L, Zaletayev D, Zuffardi O and Wood S (1995) Report of the second international workshop on human chromosome 8 mapping 1994. *Cytogenet Cell Genet* **68**: 148–155

Suzuki H, Emi M, Komiya A, Fujiwara Y, Yatani R, Nakamura Y and Shimazaki J (1995) Localization of a tumour suppressor gene associated with progression of human prostate cancer within a 1.2 Mb region of 8p22–p21.3. *Genes Chrom Cancer* **13**: 168–174

Takle LA and Knowles MA (1996) Deletion mapping implicates two tumor suppressor genes on chromosome 8p in the development of bladder cancer. *Oncogene* **12**: 1083–1087

Tanaka K, Kikuchi-Yanoshita R, Muraoka M, Konishi M, Oshimura M and Miyaki M (1996) Suppression of tumorigenicity and invasiveness of colon carcinoma cells by introduction of normal chromosome 8p12–pter. *Oncogene* **12**: 405–410

Vocke CD, Pozzatti RO, Bostwick DG, Florence CD, Jennings SB, Strup SE, Duray PH, Liotta LA, Emmert-Buck MR and Linehan WM (1996) Analysis of 99 microdissected prostate carcinomas reveals a high frequency of allelic loss on chromosome 8p12–21. *Cancer Res* **56**: 2411–2416

Yaremko ML, Wasylyshyn ML, Paulus KL, Michelassi F and Westbrook CA (1994) Deletion mapping reveals two regions of chromosome 8 allele loss in colorectal carcinomas. *Genes Chrom Cancer* **10**: 1–6

Yaremko ML, Recant WM and Westbrook CA (1995) Loss of heterozygosity from the short arm of chromosome 8 is an early event in breast cancers. *Genes Chrom Cancer* **13**: 186–191

Yaremko ML, Kutza C, Lyzak J, Mick R, Recant WM and Westbrook CA (1996) Loss of heterozygosity from the short arm of chromosome 8 is associated with invasive behavior in breast cancer. *Genes Chrom Cancer* **16**: 189–195

Yu CE, Oshima J, Fu YH, Wijsman EM, Hisama F, Alisch R, Matthews S, Nakura J, Miki T, Quais S, Martin GM, Mulligan J and Schellenberg GD (1996) Positional cloning of the Werner's syndrome gene. *Science* **272**: 258–262

Zimmermann W, Bender E, Rohde K, Reis A, Wiseman R, Futreal A, Krause H, Prokoph H, Werner S and Scherneck S (1993) Linkage analysis in German breast cancer families with early onset of the disease, using highly polymorphic markers from the chromosome 17q11–q24 region. *Am J Hum Genet* **52**: 789–791

© Cancer Research Campaign 1997